# Detection and Visualization of Heterozygosity-Rich Regions and Runs of Homozygosity in Worldwide Sheep Populations

**DOI:** 10.3390/ani11092696

**Published:** 2021-09-15

**Authors:** Alana Selli, Ricardo V. Ventura, Pablo A. S. Fonseca, Marcos E. Buzanskas, Lucas T. Andrietta, Júlio C. C. Balieiro, Luiz F. Brito

**Affiliations:** 1 Department of Nutrition and Animal Production, School of Veterinary Medicine and Animal Science (FMVZ), University of São Paulo (USP), Pirassununga 13635-900, São Paulo, Brazil; lucas.andrietta@usp.br (L.T.A.); balieiro@usp.br (J.C.C.B.); 2 Centre for Genetic Improvement of Livestock, Department of Animal Biosciences, University of Guelph, Guelph, ON N1G 2W1, Canada; pfonseca@uoguelph.ca; 3 Department of Animal Science, Federal University of Paraíba, João Pessoa 58051-900, Paraiba, Brazil; marcosbuz@gmail.com; 4 Department of Animal Sciences, Purdue University, West Lafayette, IN 47907, USA; britol@purdue.edu

**Keywords:** adaptation, artificial selection, genetic diversity, heterozygosity-enriched regions, runs of heterozygosity, *Ovis aries*

## Abstract

**Simple Summary:**

Heterozygosity-rich regions (HRRs) are regions of high heterozygosity, which can harbor important genes associated with key functional traits such as immune response and disease resilience. Runs of homozygosity (ROH) are contiguous homozygous segments of the genome, which can be informative of the population’s history, structure, demography events, and overall genetic diversity. We first detected factors impacting the identification of ROH and HRR in worldwide sheep populations, which were artificially selected for specific purposes or under natural conditions. We also identified common regions of high homozygosity or heterozygosity among these populations, where a diversity of candidate genes with distinct functions might indicate differential selection pressure on these regions in breeds with different trait expression. Moreover, we evaluated a tool commonly used in the corporate environment, making use of the business intelligence (BI) concept to support managers in the decision-making process, which allowed us to combine results from multiple analyses and create visualization schemes integrating different information. Our findings and proposed tools contribute to the development of more efficient breeding strategies and conservation of genetic resources in sheep and other livestock species.

**Abstract:**

In this study, we chose 17 worldwide sheep populations of eight breeds, which were intensively selected for different purposes (meat, milk, or wool), or locally-adapted breeds, in order to identify and characterize factors impacting the detection of runs of homozygosity (ROH) and heterozygosity-rich regions (HRRs) in sheep. We also applied a business intelligence (BI) tool to integrate and visualize outputs from complementary analyses. We observed a prevalence of short ROH, and a clear distinction between the ROH profiles across populations. The visualizations showed a fragmentation of medium and long ROH segments. Furthermore, we tested different scenarios for the detection of HRR and evaluated the impact of the detection parameters used. Our findings suggest that HRRs are small and frequent in the sheep genome; however, further studies with higher density SNP chips and different detection methods are suggested for future research. We also defined ROH and HRR islands and identified common regions across the populations, where genes related to a variety of traits were reported, such as body size, muscle development, and brain functions. These results indicate that such regions are associated with many traits, and thus were under selective pressure in sheep breeds raised for different purposes. Interestingly, many candidate genes detected within the HRR islands were associated with brain integrity. We also observed a strong association of high linkage disequilibrium pattern with ROH compared with HRR, despite the fact that many regions in linkage disequilibrium were not located in ROH regions.

## 1. Introduction

Runs of homozygosity (ROH) are contiguous homozygous segments of the genome, which can arise from the mating of two related individuals that transmit identical haplotypes to their offspring [1]. Long ROH segments are often associated with recent inbreeding, while short ROH are linked to ancient inbreeding, owing to the higher probability of recombination events occurring as the number of generations increases [2]. Thus, ROH analyses are paramount for estimating genetic diversity metrics such as ROH-based inbreeding coefficient (FROH), i.e., the ratio of the total length of an individual’s autosomal genome in ROH to the total length of the autosomal genome covered by single nucleotide polymorphism (SNP) [2]. FROH tends to be more accurate than pedigree-based inbreeding coefficients and enables the identification of specific genomic regions with greater inbreeding [3]. The identification of ROH regions also contributes to the characterization of population history, structure, and demographic events [4], and further reveals the selection signatures that are characterized by fixation of alleles under high selection pressure on a population [5,6], with a subsequent increase in homozygosity in regions around these alleles [7,8].

Runs of homozygosity have been extensively studied across many species for the quantification of inbreeding [2,3,9,10,11,12], detection of selection signatures [13,14,15], and comparison of statistical methods and identification parameters [4,16,17,18,19]. Runs of heterozygosity, most appropriately defined as heterozygosity-rich regions (HRRs) [20], represents a more recent concept [21], and is not as well described in the literature as ROH [20,22,23,24]. HRRs can also provide insights about population structure and demographic history [24], and these HRRs may harbor important *loci* for key functional traits such as immune response, survival rate, fertility, and other fitness traits [25]. To the best of our knowledge, there are no reported studies characterizing HRRs in sheep populations.

The process of sheep domestication (*Ovis aries*) started in the Fertile Crescent, approximately 11,000 years ago [26]. Nowadays, sheep are raised across the globe under divergent environmental conditions. While some breeds have been artificially selected for certain purposes (e.g., meat, milk, or wool), other populations have evolved without direct human interventions. The characterization of ROH patterns on populations selected for specific or divergent purposes could reveal genomic regions of predominant homozygosity related to the fixation of certain alleles associated with the traits under selection. Furthermore, HRRs on these populations may be an indicator of regions associated with important fitness traits [25]. Purfield et al. [27] analyzed the genome of sheep from six meat breeds to identify selection signatures using ROH and two complementary methods, Fixation Index and hapFLK [28], and observed regions under putative selection that frequently overlapped with high ROH regions. Dzomba et al. [29] also characterized the distribution of ROH islands in 13 South African sheep breeds and 31 worldwide sheep populations, which enabled the identification of common and unique ROH islands across populations.

Data visualization is a critical step in genomic data analytics for proper interpretation of the findings. Plotting results instead of looking at tabular data frequently provides additional insights into the patterns and trends of the results. Several tools have been developed to visualize genomic data, which can be challenging owing to the structure and complexity of the data [30]. Furthermore, the volume of data that usually results from independent analyses may present an additional challenge for the integration and comparison of such results, which are usually projected in distinct static images. Business intelligence (BI) is a concept used in the corporate environment to support managers in the decision-making process by enabling informed decisions based on data [31]. Distinguishing features of BI tools include the possibility of creating dynamic data visualizations and integrating distinct data sources. Consequently, BI tools provide a great opportunity for combining results from different analyses and navigating through parameters more quickly, such as by changing the exhibited chromosome or breed in a click.

In this study, we chose 17 worldwide sheep populations of eight breeds, which were intensively selected for different purposes (meat, milk, or wool), or locally-adapted breeds. Our main objectives were as follows: (1) to identify and characterize factors impacting the detection of ROH and HRR in sheep breeds selected for different breeding goals; (2) to evaluate the feasibility of using a BI tool to visualize and filter the observed results, integrating multiple types of information in a single visualization, such as ROH islands and previously-identified quantitative trait loci (QTL) or linkage disequilibrium (LD) pattern; and (3) to compare different parameters for the identification of HRR, with the aim of providing some basis for future studies of this nature in sheep and other livestock populations.

## 2. Materials and Methods

### 2.1. Genotypic Data and Quality Control

The genotypic data used in this study were made publicly available by the International Sheep Genomics Consortium [32], and downloaded from the public online WIDDE database (http://widde.toulouse.inra.fr/widde/, accessed on 15 March 2021). Genotypes were obtained using the Illumina^®^ OvineSNP50 BeadChip, and 1186 sheep from 17 worldwide populations from eight breeds were chosen for this study (Table 1). These populations were classified based on their main breeding purpose, such as meat (Lacaune, Suffolk, and Texel), milk (Churra, East Friesian Brown, and Lacaune), wool (Merino), or locally-adapted (Soay and Tibetan). The Lacaune breed comprised two populations (selected for meat or milk), while Merino, Suffolk, and Texel comprised distinct populations sampled in various countries (Table 1). The following filtering criteria were applied in the quality control (QC) for the entire dataset (all populations together): (i) markers on non-autosomal chromosomes, (ii) with missing call rate >0.05, and (iii) individuals with missing call rate >0.05 (although no individuals were removed). Data were not pruned for minor allele frequency (MAF) nor LD, as removing fixed alleles was not desired, and LD pruning has distinct effects on the detection of ROH depending on the population [19]. After QC, 46,095 markers were kept, and no individual was excluded from the dataset. QC was performed using the PLINK software v1.9 [33].

### 2.2. Data Integration and Visualization

The Business Intelligence Software Tableau V. 2020.1.14 (https://www.tableau.com, accessed on 12 September 2021) was used under the Student License to integrate data obtained from the previously described databases and analyses. As one of its key features is the ability to integrate data from multiple sources, the format of the data flat files was minimally rearranged according to the Tableau requirements, using the R software [34]. All visualizations presented in this paper were created on the BI tool, and screenshots were taken to exhibit the visualizations with certain filters applied, which were mentioned in the respective figures. The visualizations created on the Tableau platform are divided in dashboards, where one can observe the distribution of the ROHs, HRRs, and islands in the chromosomes, as well as QTLs, LD pattern, and additional information. One can zoom in the visualizations and apply different filters, such as population, chromosome, position, type of island (ROH or HRR), LD (measured as r^2^), and others. Hovering the mouse over the visualizations exhibits extra information. The entire file, with visualizations and data integrated from all the analyses, can be accessed in the Supplementary Materials section (File S1).

### 2.3. Detection of Runs of Homozygosity and Heterozygosity-Rich Regions

The R package detectRUNS [35] was used to detect both ROH and HRR, through the “Sliding Windows” method. In order to identify ROHs, the following criteria were applied: (i) the minimum number of SNPs in an ROH was 20; (ii) the minimum length of an ROH was 1000 kb (equivalent to ~20 SNPs); (iii) the minimum marker density was set to one SNP every 70 kb; (iv) the maximum gap within an ROH was 250 kb; (v) the size of the SNP window was set to 20 SNPs (according to Meyermans et al. [19], an appropriate size for the SNP window is equal to the number of SNPs in the smallest ROH); (vi) a maximum of one missing and one heterozygous SNP were allowed within an ROH and SNP window; and (vii) the window threshold of 0.05 was kept as the package default. The population average of the proportion of ROH coverage in each chromosome was calculated as (L_ROHCHR_/N)/L_CHR_, where L_ROHCHR_ is the sum of the total length of the chromosome covered by ROH of all individuals in a population, N is the number of individuals in a population, and L_CHR_ is the length of the chromosome calculated as the position of the last marker minus the position of the first marker on the chromosome in base pairs.

For the detection of HRR, different scenarios were evaluated (Table 2), and the following criteria were applied: (i) the minimum number of SNPs in a HRR ranged from 5 to 10; (ii) the minimum length of a ROH ranged from 10 to 400 kb; (iii) the minimum marker density was set to one SNP every 70 kb; (iv) the maximum gap within a HRR was 1000 kb; (v) the size of the SNP window was equal to the minimum size of a HRR; (vi) the maximum number of homozygous SNPs allowed within a HRR and SNP window ranged from one to three; (vii) the maximum number of missing SNPs allowed within a HRR and SNP window was one or two; and (viii) the window threshold was kept as the default (0.05).

### 2.4. Definition of ROH and HRR Islands

The ROH and HRR islands were defined specifically for each population, following the methodology described by Purfield et al. [27]. The R package DetectRuns [35] was used to obtain the proportion of times each SNP fell inside a run in each population, which corresponded to the locus homozygosity or heterozygosity in the respective population. In order to define the ROH and HRR islands, the top 0.999 SNPs of the percentile distribution of the locus homozygosity or heterozygosity range within each population were selected, determining different thresholds of within ROH/HRR frequency for SNPs to be included in the islands. From these top frequency SNPs within each chromosome, markers with a distance further than 250 kb from the previous (according to the max gap defined for the ROH detection) were identified as the start of a new island, and the minimum and maximum positions of SNPs in each island were assigned as the start and end of each island, respectively.

### 2.5. Identification of Regions in Strong Linkage Disequilibrium

The degree of LD was calculated as r^2^ for each population individually, using the PLINK (v1.9) software [33]. A different QC was applied to remove SNPs with MAF lower than 0.05 in each population and reduce bias in the LD estimation. Markers with a missing call rate higher than 0.1 were also excluded. The PLINK default threshold of r^2^ > 0.2 was used to select the analyses output.

### 2.6. Gene Annotation, Gene Ontology (GO), and KEGG Pathway Enrichment Analyses

The regions defined as ROH and HRR islands were annotated for gene content from the Ensembl database [36] within the coordinates using the R package GALLO [37]. These genes were further analyzed for gene ontology (GO) terms and metabolic pathway information from the Kyoto Encyclopedia of Genes and Genomes (KEGG) database [38]. The GO and KEGG pathway enrichment analyses were carried out with the R package WebGestaltR version 0.4.4 [39], using the method over-representation analysis (ORA), separately for eight subsets of genes, corresponding to the genes identified within the two types of island (ROH and HRR) and the four breed groups (meat, milk, wool, and adaptation). The model organism selected was *Homo sapiens*, as it is genetically closer to *Ovis aries*, which was not available. As the gene IDs could not be directly used to perform the analyses, only genes with gene symbol information were used. In order to obtain missing gene symbols, two methods were used: (i) the protein IDs were identified through the ovine genes’ Ensembl IDs and these protein IDs were used to obtain the gene symbols on Uniprot Kb; (ii) using the ovine Ensembl IDs, the Entrez IDs of the genes were obtained and the Rambouillet v1.0 database was used to annotate the orthologous genes in the bovine (ARS-UCD1.2) and human (GRCh38.p13) databases. Only orthologous genes with over 70% of similarity with the sheep sequence were maintained for further analyses. After matching the recovered gene symbols with the respective gene IDs, the GO and KEGG pathway enrichment analyses were performed. The GO terms and pathways were considered enriched after a multiple testing correction using a 5% false discovery rate (FDR).

### 2.7. QTL Annotation

QTL information was obtained from the Animal QTL Database [40] for the OAR 3.1 assembly, and plotted against the ROH and HRR information to identify economically relevant regions previously described in the literature within the relevant genomic regions. Only QTL identified based on SNP markers were included through the implementation of Tableau filtering criteria (map type = genome).

## 3. Results

### 3.1. Runs of Homozygosity

**Figure 1:**
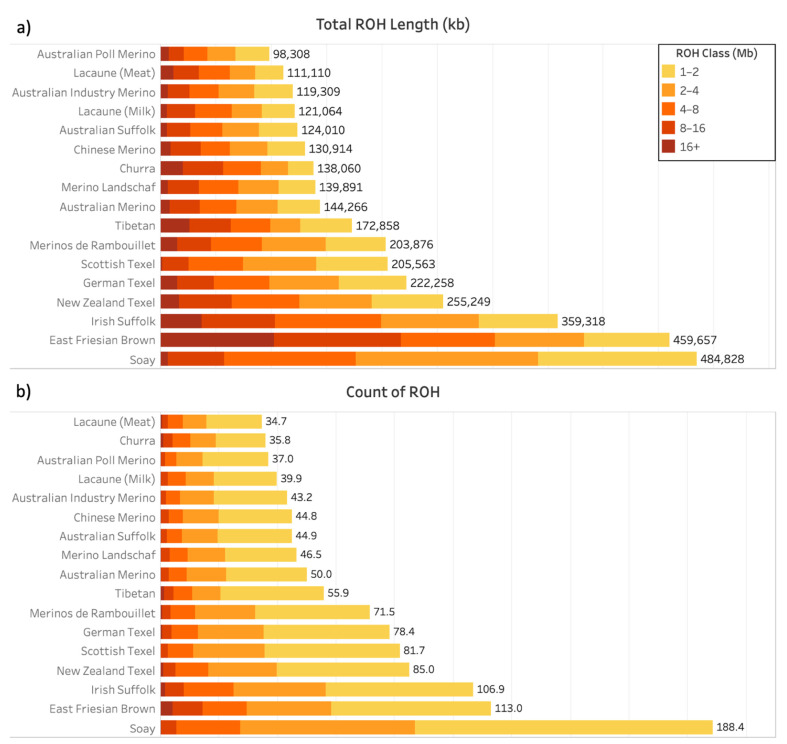
Distribution of ROH on seventeen worldwide sheep populations. (**a**) Populations average of genomic ROH length (top figure); (**b**) populations average of the number of ROH on the genome. Color scale represents the length of the runs in Mb, divided into five classes (1–2, 2–4, 4–8, 8–16, and >16 Mb).

A total of 80,639 ROH were identified in the 1186 genomic samples analyzed (67.99 ± 47.32 ROH per sample). ROH lengths ranged from 1000 kb (minimal detectable size) to 50,908 kb, identified in the East Friesian Brown population. Figure 1a shows the average length of the genome covered by ROH, and Figure 1b shows the average number of ROH for each population. ROH were classified by length, in classes of 1 to 2, 2 to 4, 4 to 8, 8 to 16, and over 16 Mb. Australian Poll Merino had the lowest average coverage of ROH (98,308 kb), and Lacaune (meat) had the lowest average number of ROH (34.7), while Soay had both the highest average coverage of ROH (484,828 kb) and the highest average number of ROH (188.4). The profile of ROH varied greatly among populations (Figure 1). The percentage of ROH in length classes for all populations is shown in Supplementary Table S1. All populations had more ROH between 1 and 2 Mb than in the other classes (Figure 1 and Table S1), and Tibetan had the highest proportion among populations (63.36%), while also having a considerable proportion of ROH longer than 16 Mb (2.13%). East Friesian Brown had the highest proportion of ROH longer than 16 Mb (3.74%), while Scottish Texel had the lowest (0.12%).

**Figure 2:**
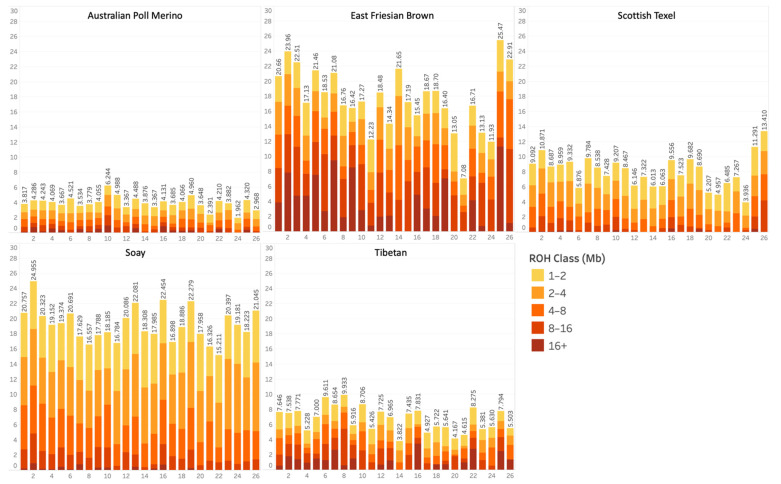
Population average of the proportion of ROH coverage in each autosomal chromosome in five selected sheep populations. The color scale represents the length of the runs of homozygosity (ROH) in Mb, divided into five classes (1–2, 2–4, 4–8, 8–16, and >16 Mb). The results for all the other populations are presented in the Supplementary Material (Tableau file).

Figure 2 provides an example of population averages of the proportion of ROH coverage in each chromosome for five populations. The results for all the other populations are presented as Supplementary Material (File S1). Australian Poll Merino and Tibetan had low percentages of ROH coverage on all chromosomes (less than 10%), while East Friesian Brown and Soay had over 20% in some chromosomes. However, ROH longer than 16 Mb were more frequently detected in the East Friesian Brown than in the Soay population (Figure 1). Scottish Texel had relatively few ROH longer than 16 Mb, whereas Tibetan, also with a low percentage of ROH coverage, had a greater amount of long ROH (mainly located in chromosomes OAR7, OAR16, OAR22, and OAR25). The distribution of ROH across chromosomes also varied among populations, as some had a significant amount of ROH coverage on a given chromosome and others had a small amount on that same chromosome.

**Figure 3:**
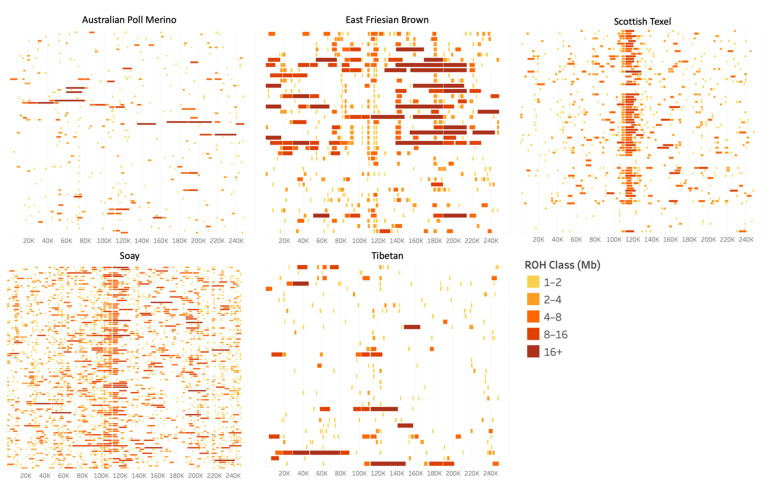
Distribution of ROH on chromosome OAR2 of five sheep populations, where each row represents an individual and the x-axis represents the position of runs in kb on the chromosome. The color scale represents the length of the runs in Mb, divided into five classes (1–2, 2–4, 4–8, 8–16, and >16 Mb). The results for all the other populations are presented in the Supplementary Material (File S1).

Plotting the ROHs of each individual enables the visualization of patterns in the distribution and size of ROH for the population, in a complementary manner to the previous figures. Figure 3 shows the distribution of ROH on OAR2 for five sheep populations, as an example (Supplementary File S1 for all populations). Hovering the mouse over each run exhibits a tooltip with specific information, such as start and end positions, number of markers, and length (File S1). New Zealand Texel, German Texel, Scottish Texel, and Soay presented clear patterns of runs on the region from 107,000 kb to 120,000 kb (Figure 3 and File S1). The different proportions of short and long ROHs are again evident when comparing East Friesian Brown and Soay populations, despite both having a similar ROH coverage on the chromosome OAR2 (Figure 2). Furthermore, we can visualize gaps between close runs as a repetitive pattern on different individuals and populations, which could be otherwise considered as single longer runs.

### 3.2. Heterozygosity-Rich Regions Detection Scenarios

For the detection of HRRs, nine scenarios with different parameters (Table 2) were tested on the entire dataset of sheep populations. In scenarios 1 to 3, the minimum length allowed for an HRR was 400, 250, and 10 kb, respectively. Scenarios 4 to 6 were similar to scenario 2, with a maximum number of homozygous allowed in an HRR and SNP window of 2, 1, and 1, respectively, and a maximum number of missing SNPs allowed in an HRR and HRR window of 2, 2, and 1, respectively. Scenarios 7 to 9 were similar to scenarios 1 to 3, with a minimum number of SNPs allowed and an SNP window of five SNPs.

**Figure 4:**
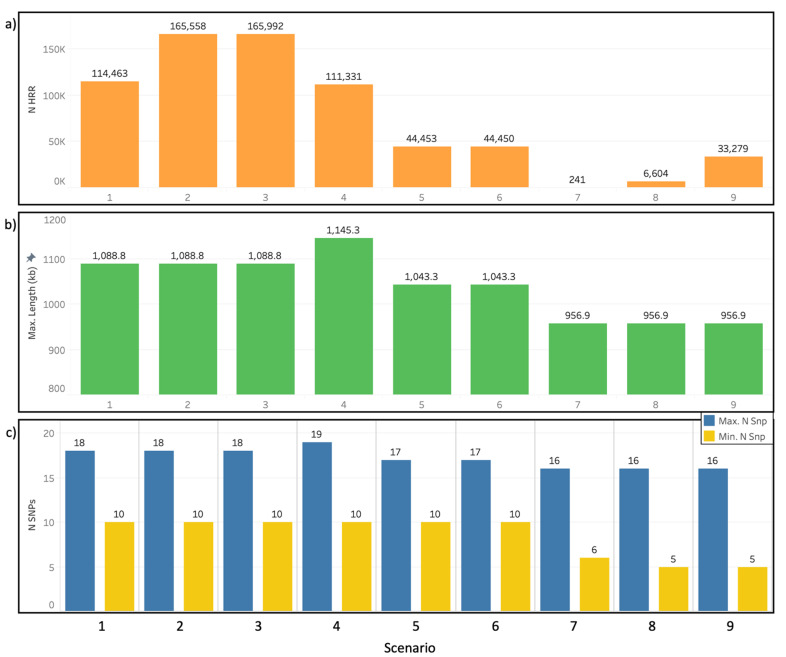
Number of heterozygosity-rich regions (HRRs) (**a**), maximum length of HRR (**b**), and maximum and minimum number of SNPs in an HRR (**c**) across nine scenarios of HRR detection, applied in a dataset of seventeen worldwide sheep populations.

Figure 4 presents the total number of HRRs (Figure 4a), the maximum length in Mb (Figure 4b), and the minimum and maximum number of SNPs within an HRR (Figure 4c) for each scenario. The number of HRRs detected increased as the minimum length allowed for an HRR was reduced, and it decreased as the minimum number of SNPs, SNP window size, maximum number of homozygous, and missing SNPs allowed were reduced (Figure 4a). The maximum length of the detected HRR was reduced when the minimum number of SNPs and the SNP window size were reduced. The impact of the number of homozygous SNPs allowed on the maximum length of the detected HRR was not so clear, and the reduction in the number of missing SNPs allowed did not affect the maximum length of the detected HRR (Figure 4b). The minimum number of SNPs in an HRR remained the same as the minimum allowed in the parameters for all scenarios, except for scenario 7, where it was one SNP larger (Figure 4c). The maximum number of SNPs in an HRR varied from 16 to 19. Scenarios with a smaller minimum number of SNPs in an HRR had shorter HRRs in terms of the maximum number of SNPs, and the effects of reducing the number of maximum homozygous allowed were not clear. Reducing the number of missing SNPs allowed had no effect on the maximum number of SNPs in an HRR (Figure 4c).

Scenario 2 was chosen to carry out the identification of HRR islands. In this scenario, the average number of HRR per animal identified across populations was 139.59, the average length was 459.894 kb, and the average of the total length was 64,198 kb. Table 3 presents each population average of total HRR length (KB) per animal and number of HRR per animal. Soay is the population with the least number of HRRs per animal (104) and total length (47,897.39 kb) per animal, and Australian Suffolk was the population with the largest number of HRRs (154) per animal and total length (70,704.99 kb) per animal (Table 3). The number of SNPs within an HRR ranged from 10 to 18, with 76.85% of the runs composed by the minimum number of SNPs (10), and only one run composed by 18 SNPs (Figure S1).

### 3.3. Presence of Linkage Disequilibrium on ROH and HRR Islands

The fact that LD plays a role in the formation and maintenance of ROH throughout generations has been reported by many authors [2,41,42,43]. In the case of ROH islands, which refer to short runs that are present in a representative portion of the population, LD may present an even stronger influence. For the purpose of investigating the association between LD and the presence of ROH and HRR islands, the islands were plotted against SNPs’ pairwise calculation of r^2^. To avoid the overlap of longer regions in LD by shorter regions within closer SNPs, we applied different filters in Tableau, restricting the minimum LD distances (length) and r^2^, thus filtering the information shown in the visualizations.

**Figure 5:**
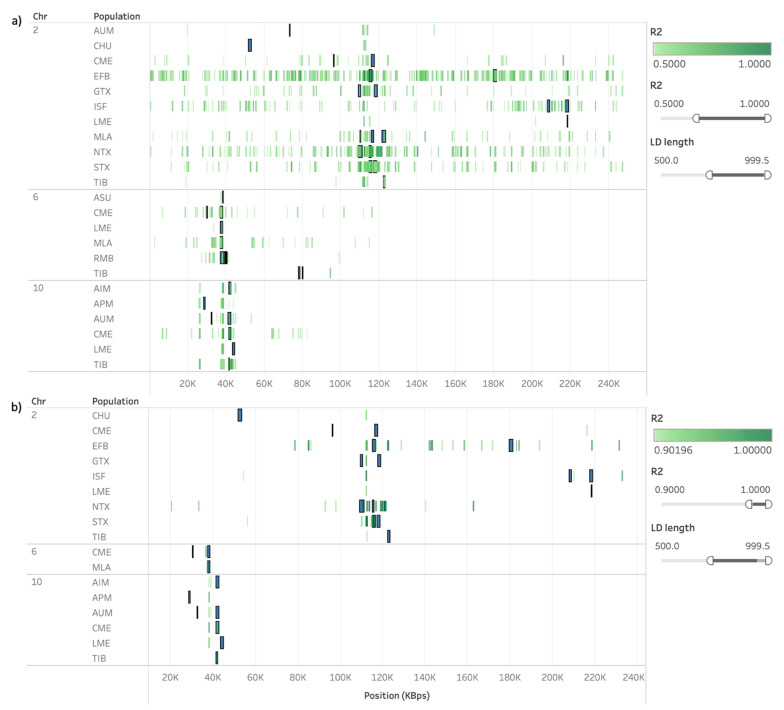
Runs of homozygosity (ROH) islands (blue) plotted against SNP pairwise calculation of r^2^ (green) in chromosomes where common regions in ROH islands were detected for more than one sheep population. Regions in linkage disequilibrium (LD) were restricted to SNP distances between 500 and 1000 kb, and r^2^ > 0.5 (**a**) or r^2^ > 0.9 (**b**). Only rows where both ROH islands were present and LD met the thresholds were presented, as a default and immutable requirement of Tableau’s visualization.

**Figure 6:**
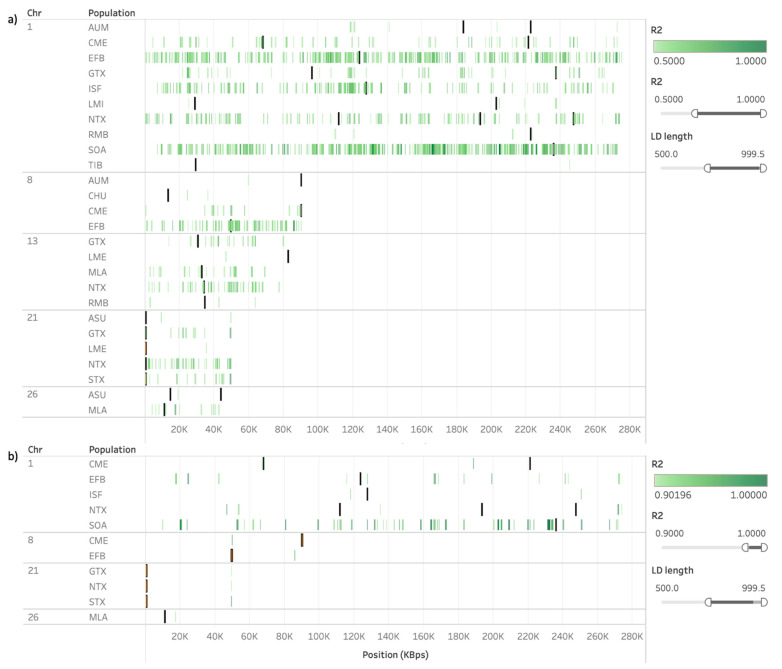
Heterozygosity-rich region (HRR) islands (orange) plotted against SNP pairwise calculation of r^2^ (green) in chromosomes where common regions in HRR islands were detected for more than one sheep population. Regions in linkage disequilibrium (LD; measured as r^2^) were restricted to SNP distances between 500 and 1000 kb, and r^2^ > 0.5 (**a**) or r^2^ > 0.9 (**b**). Only rows where both runs of homozygosity (ROH) islands were present and LD met the thresholds were presented, as a default and immutable requirement of Tableau’s visualization.

The plots of regions in LD against both ROH and HRR islands in chromosomes where common regions for more than one population were identified are presented in Figure 5 and Figure 6, respectively. We applied the same filters in both visualizations, first allowing only LD calculated between SNPs from 0.5 to 1 Mb apart and with r^2^ > 0.5, and later setting r^2^ > 0.9. In Figure 5a, we can observe that regions with relatively strong LD (r^2^ > 0.5) spanning over 500 Kb frequently overlap with ROH islands; however, not all ROH islands fall upon such regions. In Figure 5b, only regions in very strong LD (r^2^ > 0.9) are exhibited, and such regions tend to be located close to where regions in ROH islands common to more than one population were identified. In the case of HRR islands, however, their occurrence seems to be independent from the regions in LD (Figure 6a,b).

**Figure 7:**
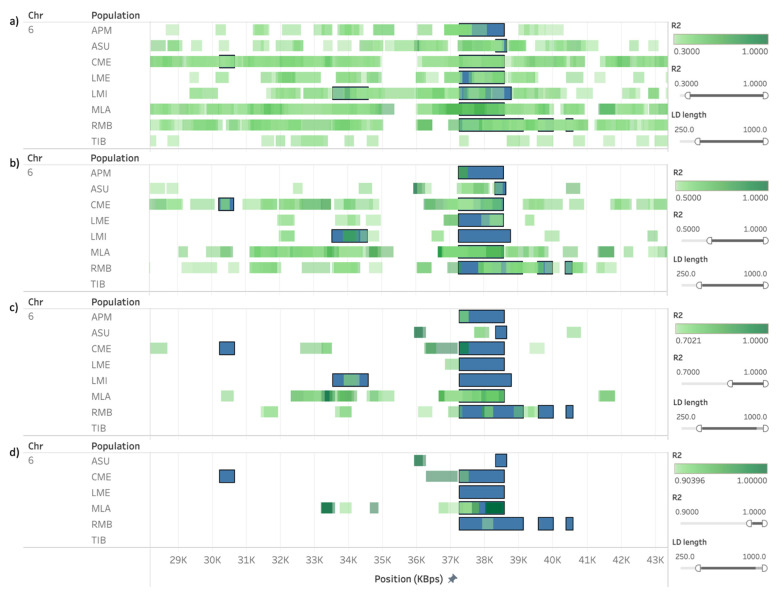
Runs of homozygosity (ROH) islands (blue) plotted against SNP pairwise calculation of r^2^ (green) in OAR6 (29–43 Mb). Regions in linkage disequilibrium (LD) were restricted to SNP distances between 250 and 1000 kb, and the r^2^ threshold was set to 0.3 (**a**), 0.5 (**b**), 0.7 (**c**), or 0.9 (**d**). Only rows where both ROH islands were present and LD met the thresholds were presented, as a default and immutable requirement of Tableau’s visualization.

We also observed the extent of LD within and close to ROH and HRR islands shared among populations. As an example, Figure 7 shows the approximation of the region between 29 and 43 Mb in OAR6, where a common ROH island was detected for up to seven sheep populations. As the r^2^ threshold increases, it is possible to visualize which regions are in stronger LD for each population. We considered a minimum length of 250 kb for the calculation of r^2^, given that the objective of this analysis was to observe blocks of LD, and the presence of small adjacent segments of LD could lead to a misrepresentation of the extent of LD in longer distances. In Figure 7a, only three populations presented at least a fraction of the detected islands free of LD blocks stronger than 0.3, showing that most of the islands in this region were under the influence of some amount of LD. However, considering LD blocks stronger than 0.5, the number of regions drastically decreased (Figure 7b), and only three of the seven populations presented LD blocks stronger than 0.9 within the islands detected on this region (Figure 7d).

### 3.4. Identification of ROH and HRR Islands and Gene Annotation

The ROH and HRR islands were defined as the SNPs within a run present on a percentage of the population above a certain threshold, defined as the 99.9% quantile of the distribution of each population. Scenario 2 was chosen as the base scenario for HRR detection. Fifty-seven ROH islands and 115 HRR islands were identified from the 17 sheep populations, after excluding HRR islands with four or less SNPs. This criterion was applied to the HRR islands in order to avoid small regions that may have resulted from the adoption of less stringent parameters for HRR detection. The list of all detected ROH and HRR islands from each population is presented in Supplementary Tables S1 and S2, respectively. The largest ROH island was found in the New Zealand Texel population, on OAR2 between 109,132 and 111,301 kb, with a length of 2,169,874 kb. The Australian Merino population had the shortest ROH (OAR4; 47,347,002–47,397,772), with a length of 50,770 kb (Table S2). The longest HRR was found in the Scottish Texel population (OAR21; 231,657–956,070), with a length of 724.413 kb, while the shortest HRR was found in the Australian Industry Merino population (OAR18; 16,667,614–16,791,775), with a length of 124,161 kb (Table S3).

There were 898 candidate genes identified within the ROH and HRR islands, from which 577 had gene symbols identified in the Ensembl database. Fifty-nine gene symbols were retrieved in total, from which one was exclusively identified from Uniprot Kb, eight were orthologous only to cattle genes, and seven to human. GO terms and pathways associated with genes identified within the ROH and HRR islands were tested for evidence of functional enrichment within the group and type of island where they were identified. Table 4 presents the GO terms enriched and the respective islands associated; all other GO and pathways can be found in Table S4. There were eleven enriched GO terms in total, five in adaptation HRR, one in milk HRR, and five in wool ROH. No pathway was enriched.

Regions in ROH or HRR islands common to two or more populations are described in Table 5 and Table 6, respectively. We identified regions in four chromosomes (OAR2, OAR6, OAR10, and OAR11) where ROH were frequent in more than one population. OAR2 harbored the greatest number of such regions (Table 5). Five regions were identified as common regions in HRR islands, and four contained at least one gene (Table 6).

### 3.5. Overlap of Known QTL with ROH and HRR Islands

**Figure 8:**
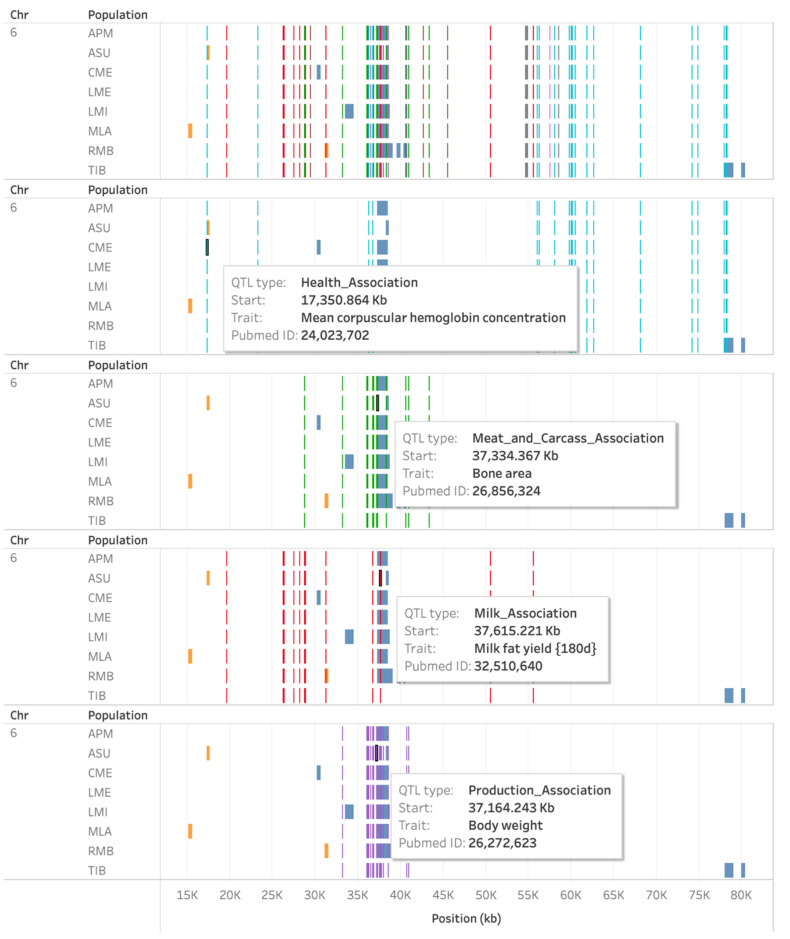
Runs of homozygosity (ROH—blue) and heterozygosity-rich regions (HRR—orange) islands on chromosome 6 plotted against different types of QTLs.

In an effort to investigate whether there is overlap of ROH or HRR islands with previously reported QTL, data from the Sheep QTL database (https://www.animalgenome.org/cgi-bin/QTLdb/OA/index, accessed on 8 August 2021) were plotted against ROH and HRR islands. Figure 8 shows a region on OAR6 between 15 and 80 Mb, harboring a few islands from eight sheep populations. On the top of the image, all types of QTL are selected. Applying QTL type filters, it becomes clearer which QTLs are overlapping with each island. Health association QTLs overlap with an HRR island in the Australian Suffolk population, as well as an ROH island from the Tibetan population and very close to other ROH islands. Meat and carcass association, milk association, and production association QTLs were found to overlap with ROH islands from the Australian Poll Merino, Australian Suffolk, Chinese Merino, Lacaune Meat, Lacaune Milk, Merino Landschaf, and Merino de Rambouillet populations. A milk association QTL also overlapped with an HRR island from the Merino de Rambouillet population. These QTLs are reported as being related to traits such as mean corpuscular hemoglobin concentration, pneumonia susceptibility, and fecal egg count (health association); bone area, fat weight in carcass, total fat area, and dressing percentage (meat and carcass association); milk fat yield in 180 days, and curd firming time (milk association); and body weight and total bone weight (production association).

## 4. Discussion

In this study, we evaluated the use of a BI Software to integrate data obtained from different databases and analyses, regarding ROH and HRR detected in worldwide sheep populations. The use of the BI concept allowed us to dynamically visualize outputs from different analyses, as well as apply filters to efficiently select specific populations, chromosomes, and parameters and focus on the interaction between the studied phenomena. Furthermore, we would like to emphasize that, although the genotypic data used in this study were collected from multiple flocks [32], and sizes of the samples were taken into consideration when selecting the populations to be included herein, any conclusions drawn from the present study should be carefully considered along with other studies that used different data sources and a considerable sample size, in order to avoid any chances of misrepresentation of the populations. Moreover, the visualization method implemented in this study could also be applied to future studies.

All sheep populations included in this study presented more than 45% of their detected ROH between 1 and 2 Mb, the shortest ROH length class defined. Many studies also reported the prevalence of ROH in the shortest length category for several sheep breeds [27,44,45,46,47]. It has been reported that modern populations of sheep usually present higher effective population sizes (Ne) and SNP diversity than cattle populations [11,27,32,48], which could be related to the prevalence of short over long ROH in sheep. Moreover, Ferenčaković et al. [17] reported that the use of low-density SNP chips for the detection of ROH may lead to an overestimation of the number of ROH shorter than 4 Mb.

Nosrati et al. [48] detected on average 50.38 ROH in individuals from the same Soay population used in the present study, which corresponds to roughly one-quarter of the runs detected herein (188.4). This divergence in the results could be attributed to the differences in the detection parameters, such as higher values of minimal number of SNPs in an ROH (40) and maximal gap between adjacent SNPs (1 Mb), as well as lower SNP density (100 kb/SNP). Our results suggest that setting a low minimal number of SNPs (20) and maximal gap (250 kb), and higher SNP density (70 kb/SNP) when using a low-density SNP chip may lead to the break of runs in regions of lower SNP density, as illustrated in Figure 3, creating an overestimation of the number of runs and an underestimation of the percentage of long runs. On the other hand, Dzomba et al. [29] applied similar parameters as in the present study, with a higher minimum number of SNPs per run (30), a lower density (100 kb/SNP), and used the method Consecutive Runs. The authors reported higher averages of the number of ROH per animal per population (considering the same populations used in the present study). We have also tested the effects of applying a 0.01 MAF filter, which had almost no effects on the overall results and caused the break of some runs. Therefore, we decided not to prune the data for MAF. Besides the Sliding Windows and Consecutive Run approaches implemented by Detect Runs, there are other software and methods that could also be used for the detection of ROH, and might lead to different results.

The distribution of ROH in length classes (Figure 1a), chromosomes (Figure 2), and positions (Figure 3) showed an obvious differentiation between populations. ROH has been shown to be non-randomly distributed across the genomes, instead they reflect the occurrence of demographic events and selection pressure for different objectives [4]. The East Friesian Brown and Soay populations showed a similar total ROH length, which leads to similar inbreeding levels. However, the percentage of long ROH was much higher in the East Friesian Brown population, indicating recent inbreeding events. The Soay population was raised in isolation on the Soay Island for hundreds of years [49], and inbreeding was probably frequent when the first individuals arrived on the island, hence the high number of small runs. The three Texel and the two Lacaune populations presented similar averages of total length and number of ROH within each breed, while the two Suffolk and the six Merino populations showed a significant divergence on these metrics (Figure 1), which might indicate that the processes of selection in different countries can be more differentiated for some breeds than for others.

Few studies have been conducted with the aim of characterizing HRR in livestock, and only one has attempted to identify factors impacting HRR detection, using a low-density SNP chip [23]. Furthermore, most of the studies on HRRw used high-density SNP chips [21,22,24], which have been shown to require other parameters than low density SNP chips for ROH detection [17,41]. The same is most likely true for the identification of HRRs. In this study, we set the minimum number of SNPs within an HRR at 5 or 10, which is lower than the number used for ROH (15) because HRRs are usually reported as being shorter than ROH [23]. The same difference in the parameters was observed in other studies [20,21,24]. We observed that changing the minimal number of SNPs and window size from 10 to 5 did not increase the number of HRRs detected; in fact, the number and length of HRRs detected decreased. This could be related to the fact that we used the sliding window approach, and the reduction in the window size may have had an interaction with the other parameters, such as number of missing and homozygous SNPs allowed, causing the HRR to break even shorter. We also tested allowing different numbers of homozygous (1 to 3) and missing (1 or 2) SNPs within an HRR. Biscarini et al. [23] reported that allowing only one homozygous SNP reduced the number of detected HRR and increased its average size when compared with allowing two homozygous SNPs, while increasing this number to up to five caused both metrics to increase. We observed a similar effect in our data—when reducing the number of homozygous allowed from three to two, the number of HRR detected was reduced and the length increased, and reducing it to one caused both metrics to decrease.

Scenario 2 was chosen as the best scenario for the detection of HRR islands, for presenting a high number of HRRs and a satisfactory maximum HRR length, when compared with the other scenarios. The average number of HRRs detected per animal (139.59) was higher than that detected by other authors in turkey (57.80), cattle (9.87), and horse (52.17) populations [20,23,24], and similar to the number detected by Ferenčaković et al. [22] in a cattle population (122.52). Most of these studies reported the detection of higher numbers of ROH than HRR; however, our results showed the opposite. We hypothesized two reasons: (1) misadjustment of parameters for the detection of HRR, or (2) the sheep genome of the populations analyzed presents small and frequent HRR. Therefore, further research is needed as a means to further test these hypotheses, using different parameters and methods for the detection of HRRs. The use of a higher density SNP chip could also provide further insights.

Kijas et al. [32] reported the inbreeding coefficient (F) calculated for each of the populations used in this study, and the populations with the lowest F, such as Chinese Merino (0.08), Australian Suffolk (0.08), and Australian Poll Merino (0.09), presented higher average numbers of HRRs and total HRR length per individual (Table 3), while populations with the highest F, such as Soay (0.33), East Friesian Brown (0.26), and Irish Suffolk (0.22), presented the lowest HRR metrics (Table 3). When comparing the average numbers and total length of ROH (Figure 1) and HRR (Table 3) for the populations, a negative correlation between them was also observed.

With the purpose of investigating the occurrence of LD within ROH and HRR islands, we plotted the results from pairwise SNP calculations of r^2^ against the islands, applying filters on Tableau of minimal LD length and r^2^ values. This approach was shown to be effective because, differently from other studies, the LD values could be presented directly and not through summarizations such as average r^2^ per bins of distance (e.g., Mastrangelo et al. [50]). Moreover, when observing LD within the islands, we could identify the minimum amount of LD present and visualize the location of the LD blocks within the islands, instead of calculating the r^2^ between the first and last SNPs (e.g., Mastrangelo et al. [51] and Purfield et al. [27]), which could overshadow the presence of stronger LD between closer SNPs within the island.

Using the approach described above, we observed that most of the regions in ROH islands identified in more than one population (Table 5) were located in regions with some extent of LD (r^2^ > 0.2), with few exceptions where no LD was detected in some portion of the islands, even when allowing the minimal LD length (0 bp) and r^2^ (0.2). The presence of stronger LD within the islands varied depending on the chromosome and the population, and some populations showed more overall LD than others. Interestingly, some regions showed a strong LD (r^2^ > 0.9) in blocks over 250 Mb long across many populations, such as the region around 112 Mb in OAR2, and no islands were identified in such regions. New Zealand Texel presented LD over 0.9 in blocks within the region of 118,497–121,331 kb, and no island within the region (File S1). These findings could indicate poor identification of ROH islands, but also that the presence of strong LD in certain regions does not always result in an increase in homozygosity.

The regions detected as ROH islands for two or more sheep populations in the present study spanned across populations selected for different purposes. Abied et al. [52], using data from the OARv4.0 assembly, detected candidate regions on OAR2, OAR6, and OAR10 for five Chinese sheep breeds. Gorssen et al. [53], analyzing 100 populations from the same public database used in this study, identified islands in the same region of OAR6 (around 38 Mb) identified herein, for 15 populations. This region was a common island for four of the six merino populations we analyzed, including the Chinese Merino and the two Lacaune populations (meat and milk). He et al. [46] also identified an ROH hotspot on this region in a Chinese Merino population, and reported the influence of *NCAPG/LCORL*, genes associated with calving ease and fetal growth in cattle [54,55], body size in mammals [56,57,58], and reduced subcutaneous fat thickness in cattle [58]. A few QTLs within or very close to the region were associated with body weight (7), bone area (2), and milk fat yield. Taken together, these results suggest that this region on OAR6 is important for multiple traits, which could be beneficial for meat, wool, and milk production.

The region from 109 Mb to 119 Mb on OAR2 harbored ROH islands from six different populations, including breeds selected for meat, milk, and wool. Moreover, a great number of genes with distinct functions were observed within this region, such as *CLCN3,* a gene involved in several basic cellular functions, and that was shown to reduce the inflammatory response induced by a high-fat diet in mice [59]; *HPF1,* associated with early embryonic development in zebrafish [60]; *PMS1* and *ERCC3,* identified as candidate genes in a genomic footprint for dryland stress adaptation in Egyptian fat-tail sheep [61]. Purfield et al. [27] reported the region between 115.48 and 126.34 Mb on OAR2 as the ROH hotspot with the most occurrences and as under putative selection in breeds selected for meat (i.e., Texel), but not for Suffolk. In our study, the Texel and the Suffolk populations did not share common islands, in agreement with Purfield et al. [27], who reported a significant differentiation between these breeds. The QTLs observed within 109 Mb and 119 Mb on OAR2 were mostly related with horn type (21); meat color (1) and texture (1); and health traits, such as fecal egg count, platelet count, mean corpuscular volume, and hemoglobin level. These results also indicate that a variety of traits are impacted by this region, thus harboring ROH islands for different selection groups.

We identified three genes (*BIN1*, *MYO7B*, and *GAS7*) in common ROH islands that were associated with terms related to muscle development and enriched in the wool group: Actin Cytoskeleton (GO:0015629) Actin Binding (GO:0003779), Contractile Fiber (GO:0043292), and Motor activity (GO:0003774). *BIN1* and *MYO7B* were detected in a region in OAR2 shared by Chinese Merino, Merino Landschaf, and Scottish Texel. *B1N1* is involved in muscle cell differentiation [62]. It was reported by Purfield et al. [27] as a candidate gene in Texel, and by Al Kalaldeh et al. [63] as a candidate gene in a GWAS study for parasite resistance in Australian sheep. *GAS7* was identified in a different region, located on OAR11 and shared by Australian Industry Merino and Australian Suffolk. This gene is expressed in the central nervous system and associated with motor activity and muscle fiber composition [64].

Furthermore, Australian Industry Merino and Australian Suffolk shared a region on OAR11 where two genes (*PIK3R5* and *STX8*) were previously detected in a putative selection region in Swiss sheep [45], and are associated with body size [65,66]. *DHRS7C* and *NTN1*, also detected within this region, were reported as being related to enhanced muscle performance [67] and body size [65,66], respectively. QTLs detected within this region are associated with body height, average daily gain, milk yield, and milk fat yield. According to Safari et al. [68], there are moderate positive correlations between live weight at various ages and wool traits. They suggested that a greater need for both wool and meat products led sheep breeders to combine these two traits, as well as quality and disease resistance, into their breeding objectives. Other authors also endorsed the selection of Merino flocks for meat and carcass traits [69,70] and disease resistance [71]. Therefore, we suggest that the need to improve a variety of traits led breeds with distinct selection purposes to present a higher homozygosity in certain common regions, described herein as well as in other studies, where these distinct traits would be improved.

No gene nor QTL were detected within the region shared by Australian Industry Merino, Australian Merino, Chinese Merino, and Tibetan populations in OAR11 (41,526–42,049 kb), which may indicate the need for better annotation of the sheep genome, or that this region contains distal regulatory elements, such as silencers or enhancers. Fewer common genomic regions were identified in HRR islands than in ROH islands. From those, two regions contained identified genes. Australian Merino, Australian Poll Merino, and Chinese Merino shared a region in OAR8 (89,939–90,351 kb), which contains *TCTE3*, a gene previously described as a candidate influencing congenital diaphragmatic hernia [72] and sperm motility and morphology [73]. Three protein-coding genes (*ERMARD, PHF10,* and *WDR27*) detected within this region were previously reported in a study about structural brain abnormalities in humans, and only *ERMARD* and *PHF10* were considered as plausible candidates [74]. Furthermore, it was reported that heterozygous variants in *ERMARD (C6orf70)* are associated with brain anomalies and syndromic dominant forms of periventricular nodular heterotopia in humans [75,76]. *WDR27* was also detected as a candidate for insomnia [77].

The other common region in HRR with detected genes was identified on OAR21 (400–926 kb) and was shared by Australian Industry Merino, Australian Suffolk, German Texel, Lacaune (meat), New Zealand Texel, and Scottish Texel. *CEP295* and *MED17*, genes identified within this region, are responsible for building centrioles [78,79] and for the transcriptional activation of lipogenic genes in response to insulin [80], respectively. *VSTM5,* also identified within this region, codes a protein responsible for the regulation of neuronal morphogenesis and migration during cortical development in the brain [81].

A common region in HRR was observed in OAR13 (34,513–34,530 kb) for Merino de Rambouillet and New Zealand Texel. Despite no annotated genes being detected within this region, two QTLs were identified nearby. A QTL for milk fat yield was detected within the region in HRR island exclusive of New Zealand Texel (34,254.2–34,530.07 kb), and a QTL for average daily gain was detected outside, but near the HRR island detected in the Merino de Rambouillet (34,513.4–34,887.99 kb). A QTL for milk fat yield was also identified near an HRR island detected in Australian Suffolk, Churra, and Lacaune (milk) in OAR26 (43,609–44,004 kb).

## 5. Conclusions

In this study, we detected ROH and HRR islands in worldwide sheep populations. The parameters applied for the identification of ROH resulted in an inflation in the number of short ROH owing to the fragmentation of longer ROH. We also characterized HRRs, which had not yet been reported in sheep, and provided comprehensive knowledge about the effects of changing the parameters for HRR detection using the Sliding Windows approach. Our findings suggest that HRRs in sheep are small and frequent, and further studies using a higher density SNP chip are suggested. Regions in high LD were more closely located from ROH than HRR islands, and many regions in LD were not in ROH. Candidate genes and QTLs identified within common regions in ROH islands for different populations were related to a variety of production traits (e.g., body wight, milk fat yield, and meat color), while genes identified within common HRR islands may play a fundamental role in the survival of these individuals, as many of them are involved in brain integrity. The integration and visualization of genomic data from worldwide sheep populations, after applying filters to highlight the key results from independent analyses, allowed us to better understand structure, distribution, and LD pattern in ROH and HRR regions, as well as to identify candidate genes, QTLs, and related phenotypes.

## Figures and Tables

**Table 1:** Number of samples and traits of interest of worldwide sheep populations.

Population	Abbreviation	N	Main Trait of Interest
Australian Industry Merino	AIM	88	Wool
Australian Poll Merino	APM	98	Wool
Australian Merino	AUM	50	Wool
Chinese Merino	CME	23	Wool
Merino Landschaf	MLA	24	Wool
Merinos de Rambouillet	RMB	102	Wool
Australian Suffolk	ASU	109	Meat
Irish Suffolk	ISU	55	Meat
German Texel	GTX	46	Meat
New Zealand Texel	NTX	24	Meat
Scottish Texel	STX	80	Meat
Lacaune (Meat)	LME	78	Meat
Churra	CHU	120	Milk
East Friesian Brown	EFB	39	Milk
Lacaune (Milk)	LMI	103	Milk
Soay	SOA	110	Adaptation
Tibetan	TIB	37	Adaptation

**Table 2:** Scenarios with different parameters for the detection of heterozygosity-rich regions (HRRs).

Scenario	Consecutive SNPs	Density (SNP/kb)	Max Gap (kb)	Min Length (kb)	N Hom	N Miss	Hom Window	Miss Window	SNP Window
1	10	1/70	1000	400	3	2	3	2	10
2	10	1/70	1000	250	3	2	3	2	10
3	10	1/70	1000	10	3	2	3	2	10
4	10	1/70	1000	250	2	2	2	2	10
5	10	1/70	1000	250	1	2	1	2	10
6	10	1/70	1000	250	1	1	1	1	10
7	5	1/70	1000	400	3	2	3	2	5
8	5	1/70	1000	250	3	2	3	2	5
9	5	1/70	1000	10	3	2	3	2	5

N hom = maximum number of homozygous SNPs allowed in an HRR, N miss = maximum number of missing SNPs allowed in an HRR, Hom window = maximum number of homozygous SNPs allowed in a window, Miss window = maximum number of missing SNPs allowed in a window, SNP window = number of SNPs in a window.

**Table 3:** Averages of total heterozygosity-rich regions’ (HRRs) length (KB) and number of HRRs per animal in 17 worldwide sheep populations.

Population	Total HRR Length (KB)	N HRR
Australian Industry Merino	67,506.67	147
Australian Poll Merino	69,169.01	150
Australian Merino	68,264.90	148
Chinese Merino	68,882.00	149
Merino Landschaf	68,950.22	150
Merinos de Rambouillet	64,572.10	140
Australian Suffolk	70,704.99	154
Irish Suffolk	56,993.71	125
German Texel	63,698.25	139
New Zealand Texel	63,928.56	138
Scottish Texel	64,990.93	141
Lacaune (Meat)	67,812.41	148
Churra	66,742.96	145
East Friesian Brown	54,900.29	119
Lacaune (Milk)	67,166.13	147
Soay	47,897.39	104
Tibetan	55,394.81	122

**Table 4:** Gene ontology (GO)-enriched terms and the respective types of island where associated genes were identified.

Accession	Name	*p*-Value	FDR	Genes	Ontology
Adaptation HRR					
GO:0061134	Peptidase regulator activity	2.31 × 10^−9^	6.52 × 10^−7^	*SERPINA10, SERPINA6, SERPINA11, SERPINA12, SERPINA5, SERPINA4, SERPINA3*	Molecular Function
GO:0051346	Negative regulation of hydrolase activity	2.64 × 10^−9^	2.25 × 10^−6^	*PPP4R4, SERPINA10, SERPINA6, SERPINA11, SERPINA12, SERPINA5, SERPINA4, SERPINA3*	Biological Process
GO:0045861	Negative regulation of proteolysis	1.63 × 10^−8^	6.94 × 10^−6^	*SERPINA10, SERPINA6, SERPINA11, SERPINA12, SERPINA5, SERPINA4, SERPINA3*	Biological Process
GO:0004857	Enzyme inhibitor activity	9.18 × 10^−8^	1.29 × 10^−5^	*SERPINA10, SERPINA6, SERPINA11, SERPINA12, SERPINA5, SERPINA4, SERPINA3*	Molecular Function
GO:0052547	Regulation of peptidase activity	7.19 × 10^−8^	2.04 × 10^−5^	*SERPINA10, SERPINA6, SERPINA11, SERPINA12, SERPINA5, SERPINA4, SERPINA3*	Biological Process
Milk HRR					
GO:0045111	Intermediate filament cytoskeleton	1.18 × 10^−4^	2.04 × 10^−2^	*KRTAP15-1, KRTAP13-3, KRTAP13-4, KRTAP27-1, KRTAP24-1*	Cellular Components
Wool ROH					
GO:0003774	Motor activity	3.65 × 10^−6^	1.03 × 10^−3^	*MYH10, MYH13, MYH8, MYH4, MYH1, MYH2, MYH3, MYO7B*	Molecular Function
GO:0015629	Actin cytoskeleton	8.78 × 10^−6^	1.51 × 10^−3^	*MYH10, GAS7, MYH13, MYH8, MYH4, MYH1, EEF1A1, PXN, MYH2, MYH3, MYO7B, BIN1, CTNNA1, PDLIM5*	Cellular Components
GO:0003779	Actin binding	2.51 × 10^−5^	3.54 × 10^−3^	*MYH10, GAS7, MYH13, MYH8, MYH4, MYH1, MYH2, MYH3, MYO7B, BIN1, CTNNA1, PDLIM5*	Molecular Function
GO:0043292	Contractile fiber	2.38 × 10^−4^	2.05 × 10^−2^	*MYH13, MYH8, MYH4, MYH1, MYH2, MYH3, SCO1, BIN1*	Cellular Components
GO:0005516	Calmodulin binding	3.29 × 10^−4^	3.09 × 10^−2^	*MYH10, MYH13, MYH8, MYH4, MYH1, MYH2, MYH3*	Molecular Function

FDR: false discovery rate.

**Table 5:** Common genomic regions in ROH islands for two or more worldwide sheep populations and genes identified within.

Populations	OAR	Start (bp)	End (bp)	Genes
GTX, NTX	2	109,487,038	110,606,314	*CLCN3, HPF1, MFAP3L, NEK1, U6*
GTX, MLA, NTX	2	110,252,253	110,606,314	*CLCN3, HPF1, NEK1, U6*
EFB, NTX, STX	2	115,008,897	115,912,934	*-*
EFB, MLA, STX	2	116,108,127	116,500,683	*AMMECR1L, GLRX, POLR2D, SAP130, UGGT1*
CME, EFB, MLA, STX	2	116,166,895	116,500,683	*AMMECR1L, GLRX, POLR2D, SAP130*
CME, MLA	2	116,166,895	117,341,513	*AMMECR1L, BIN1, CYP27C1, ERCC3, GLRX, IWS1, LIMS2, MAP3K2, MYO7B, POLR2D, PROC, SAP130, SFT2D3, U6, WDR33*
CME, MLA, STX	2	117,158,936	117,341,513	*BIN1, U6*
CME, STX	2	117,158,936	117,573,048	*BIN1, NAB1, U6*
GTX, STX	2	117,795,421	118,745,085	*ANKAR, ASDURF, C2orf88, GDF-8, HIBCH, ORMDL1, OSGEPL1, PMS1, SLC40A1*
ISF, LME	2	218,427,149	218,592,494	*-*
APM, CME, LME, LMI, MLA, RMB	6	37,254,883	38,580,198	*DCAF16, LCORL, NCAPG*
APM, ASU, CME, LME, LMI, MLA, RMB	6	38,310,652	38,580,198	*-*
AIM, AUM, CME, TIB	10	41,526,980	42,049,970	*-*
AIM, ASU	11	27,877,134	28,779,375	*5S_rRNA, CFAP52, DHRS7C, GAS7, GLP2R, GSG1L2, NTN1, PIK3R5, RCVRN, STX8, USP43*

AIM: Australian Industry Merino; ASU: Australian Suffolk; APM: Australian Poll Merino; AUM: Australian Merino; CME: Chinese Merino; EFB: East Friesian Brown; GTX: German Texel; ISF: Irish Suffolk; LME: Lacaune Meat; LMI: Lacaune Milk; MLA: Merino Landschaf; NTX: New Zealand Texel; RMB: Merino de Rambouillet; STX: Scottish Texel; TIB: Tibetan.

**Table 6:** Common genomic regions and candidate genes in HRR islands for two or more worldwide sheep populations.

Population	OAR	Start (bp)	End (bp)	Genes
AIM, AUM, RMB	1	222,876,890	223,202,691	*5S_rRNA*
AUM, APM, CME	8	89,939,786	90,351,468	*C6orf12, ERMARD, PHF10, TCTE3, WDR27*
RMB, NTX	13	34,513,412	34,530,043	*-*
AIM, ASU, GTX, LME, NTX, STX	21	400,938	926,701	*C11orf54, CEP295, MED17, SMCO4, SNORA25, SNORA8, TAF1D, VSTM5*
ASU, CHU, LMI	26	43,609,868	44,004,281	*U6*

AIM: Australian Industry Merino; ASU: Australian Suffolk; APM: Australian Poll Merino; AUM: Australian Merino; CHU: Churra; CME: Chinese Merino; GTX: German Texel; LME: Lacaune Meat; LMI: Lacaune Milk; NTX: New Zealand Texel; RMB: Merino de Rambouillet; STX: Scottish Texel.

## Data Availability

All the data is available in the main text or Supplementary Materials section.

## Supplementary Materials

The following are available online at https://www.mdpi.com/article/10.3390/ani11092696/s1, Figure S1. Number of heterozygosity-rich regions (HRRs) composed of different numbers of SNPs. Table S1. Percentage of runs of homozygosity (ROH) in length classes for seventeen worldwide sheep populations. Table S2. Runs of homozygosity (ROH) islands from seventeen worldwide sheep populations. Table S3. Heterozygosity-rich region (HRR) islands from seventeen worldwide sheep populations. Table S4. Gene ontology (GO) terms and pathways associated with genes identified within runs of homozygosity (ROH) and heterozygosity-rich region (HRR) islands from different sheep groups. File S1. Tableau file with all visualizations included in this study.
