# Supplementary figures and images for: Detection and Visualization of Heterozygosity-Rich Regions and Runs of Homozygosity in Worldwide Sheep Populations
Source: Animals (Basel). 2021 Sep 15;11(9):2696. doi: 10.3390/ani11092696 (PMC8472390; doi:10.3390/ani11092696)

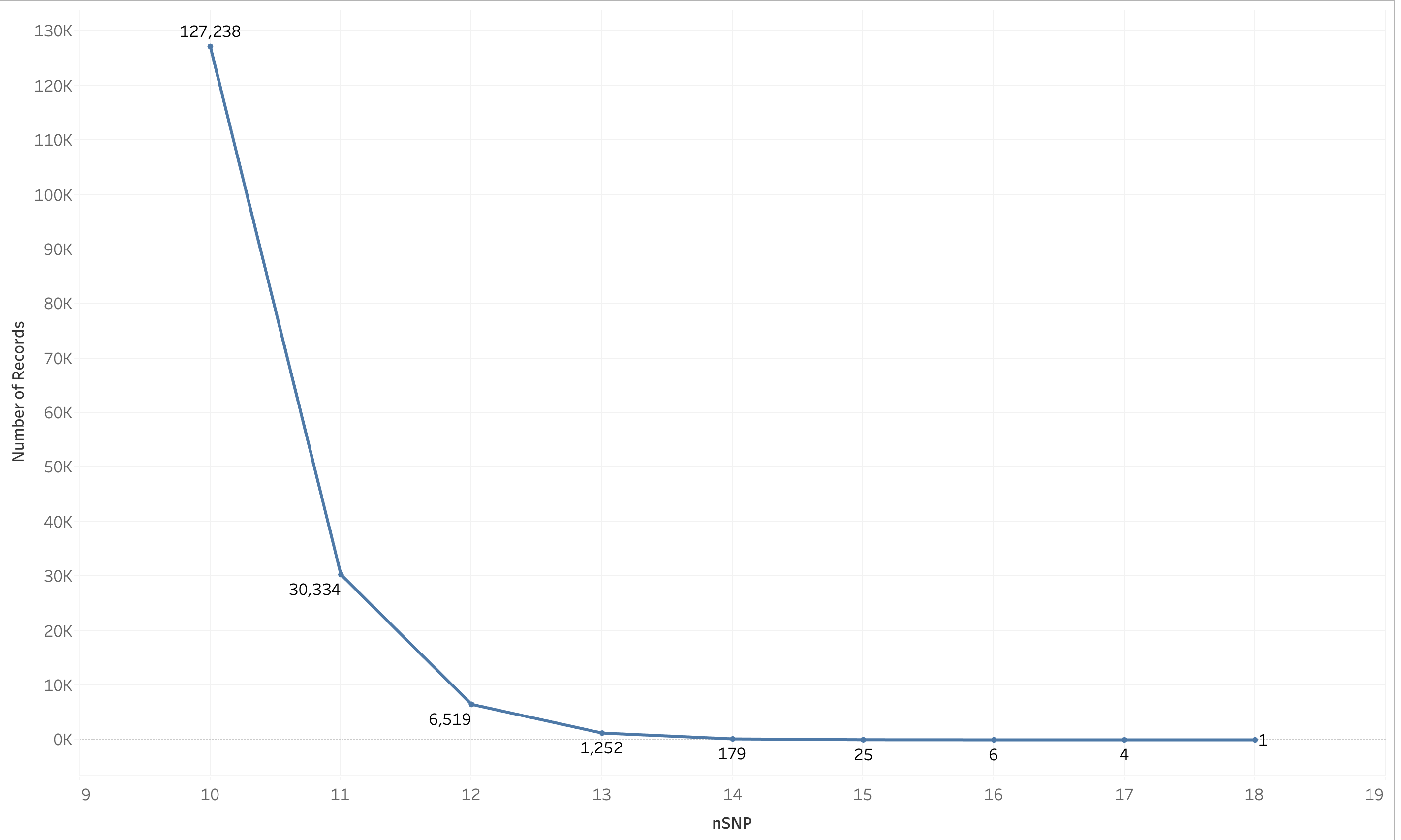

Supplement: Supplementary file 1 [file animals-11-02696-s001.zip › Figure_S1.png]
